# Endoscopic Third Ventriculostomy: A Single-Center Experience in Greece

**DOI:** 10.1055/s-0040-1708864

**Published:** 2020-05-07

**Authors:** V. Kontojannis, E. Papadopoulos, J. Ydreos, D. Isaakidis, M.M. Gavra, E.J. Boviatsis

**Affiliations:** 1 2nd Department of Neurosurgery, “Attikon” University Hospital, National and Kapodistrian University, Athens Medical School, Athens, Chaidari, Greece; 2 Department of CT and MRI Imaging, “Agia Sofia” Children's Hospital, Athens, Greece

**Keywords:** endoscopic third ventriculostomy, treatment of adult hydrocephalus, obstructive hydrocephalus

## Abstract

Endoscopic third ventriculostomy is an important tool in the treatment of various forms of adult hydrocephalus, and its use is evolving over the past years, proving in many cases more effective than the more traditional ventriculoperitoneal shunts. We present the experience from our department while comparing the results and complications with the international literature.

Currently, the creation of a foramen between the third ventricle and the interpedicular cistern under the direct guidance of an endoscope is a well-established surgical technique called endoscopic third ventriculostomy (ETV). The management of obstructive hydrocephalus using ETV is becoming the treatment of choice, expanding also in cases of communicating hydrocephalus such as post-traumatic. 1

It was in 1923 when W. J. Mixter achieved to open the third ventricle into the interpedicular cistern for the first time in a 9-month-old infant 2 suffering from obstructive hydrocephalus. He did so by using an urethroscope to visualize the floor of the third ventricle, which he then perforated using sound. Different attempts have taken place since then, but the technique in its current form was introduced in the 1990s. 3 4 Currently, there are two treatment methods for hydrocephalus patients: cerebrospinal fluid diversions techniques (shunts) and ETV. In the current project, we present our experience in managing patients suffering from hydrocephalus performing ETV and we compare our results and complications with the international literature.

## Materials and Methods

During the last 3 years from 2016 to 2019, at 2 ^nd^ Department of Neurosurgery at ‘Attiko’ University Hospital of Athens, Greece, 20 adult patients underwent ETV due to various causes ( Table 1 ).

**Table 1:** Causes of hydrocephalus

Causes	Number of patients	Preoperative symptoms
**Infection** ** ( Fig. 1 ) **	3 (15%)	Gait disorder, headache, impaired level of consciousness
**Post-traumatic**	1 (5%)	Vegetative state
**Tumor**	5 (25%)	Impaired level of consciousness
**Aqueduct stenosis** ** ( Fig. 2 ) **	2 (10%)	Incontinence, dementia, gait disorder
** Normal pressure hydrocephalus ( Fig. 3 ) **	5 (25%)	Incontinence, dementia, gait disorder
**Intraventricular bleeding**	2 (10%)	Coma
** Cavernoma ( Fig. 4 ) **	2 (10%)	Impaired level of consciousness
**Total**	20 (100%)	

**Fig. 1:**
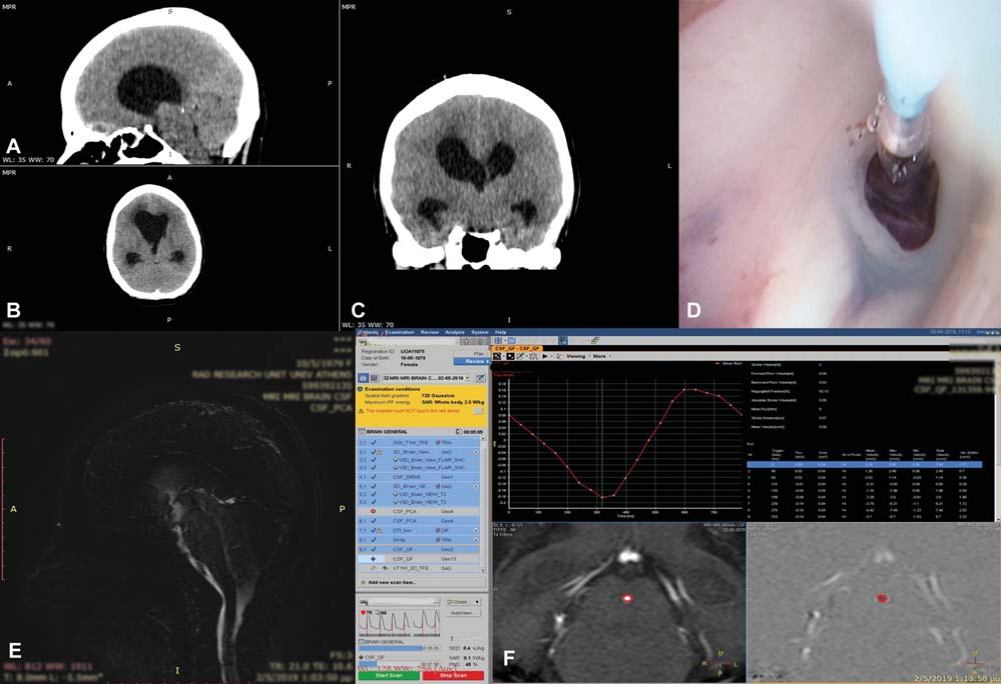
Preoperative CT scan: ( **A** ) sagittal plane, ( **B** ) axial plane, ( **C** ) coronal plane images, ( **D** ) intraoperative endoscopic image of the endoscopic third ventriculostomy created using a light-touch balloon catheter, ( **E** ) sagittal postop MRI showing the CSF flow through the ventriculostomy, and ( **F** ) MRI-CSF through the ventriculostomy showing sufficient velocity flow). CSF, cerebrospinal fluid; CT, computed tomography; MRI, magnetic resonance imaging.

**Fig. 2:**
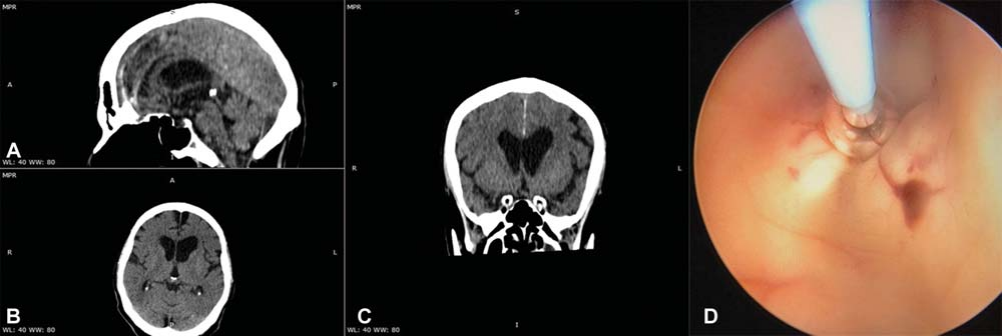
Patient suffering from hydrocephalus caused by stenosis. **A** : sagittal plane, **B** : axial plane, **C** : coronal plane postoperative CT scan, and **D** : intraoperative image of the creation of the ventriculostomy using the balloon catheter at the insertion area above the mamillary bodies.

**Fig. 3:**
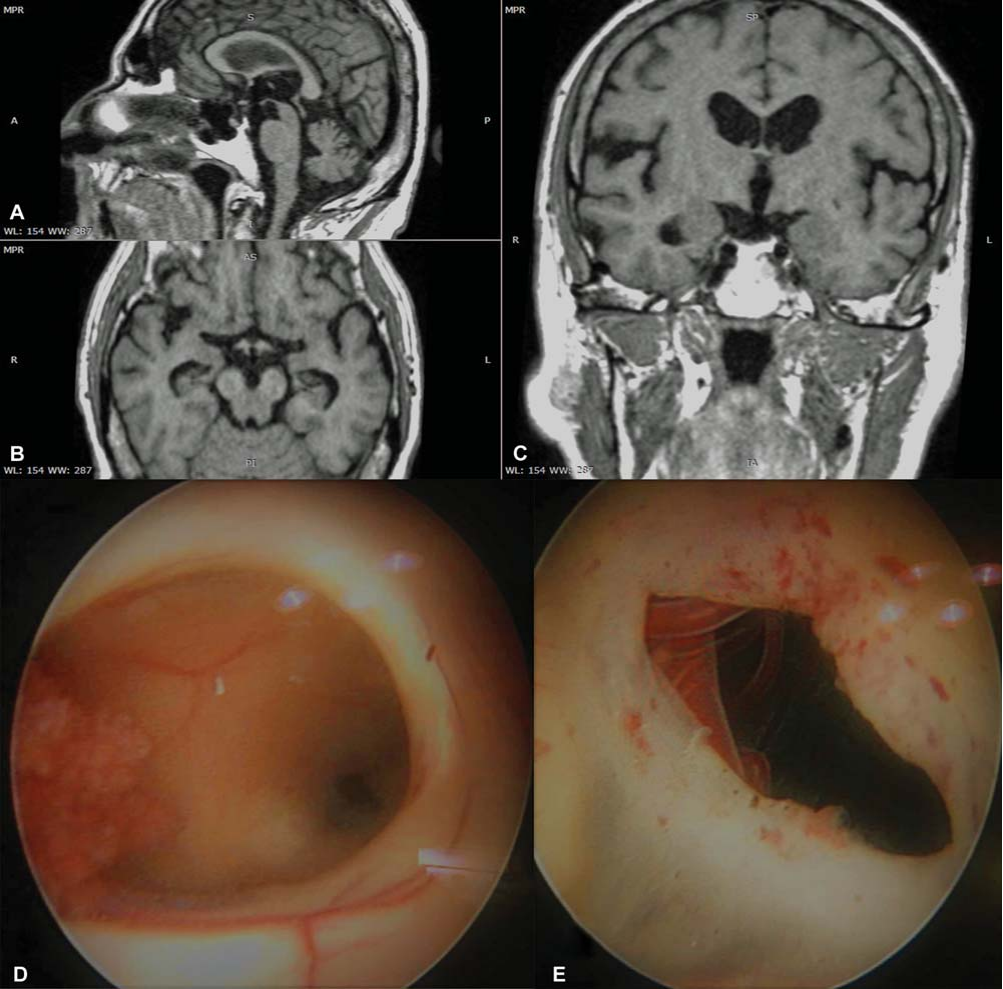
Patient suffering from normal pressure hydrocephalus. **A** : sagittal, **B** : axial, **C** : coronal postoperative MRI scan, **D** : intraoperative image of the endoscopic third ventriculostomy stoma at the floor of the 3rd ventricle, and **E** : endoscopic image through the ventriculostomy showing the basal artery and it's perforating branches to the brain stem.

**Fig. 4:**
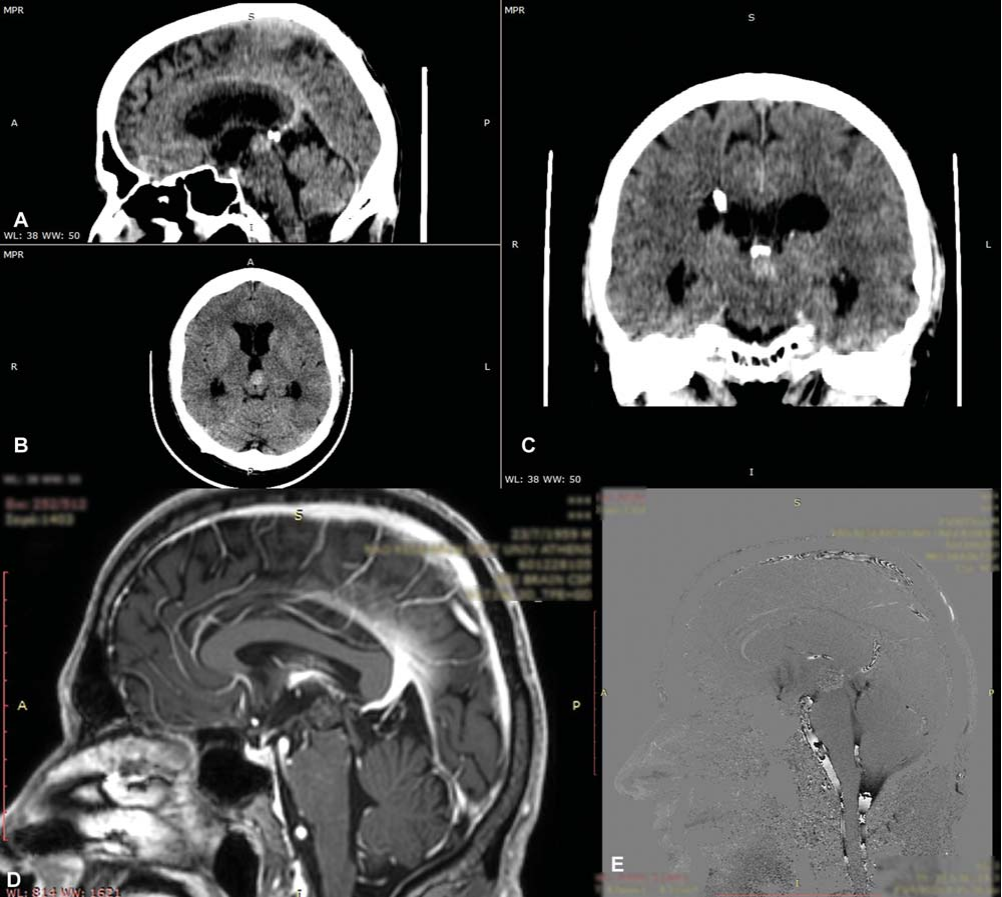
Patient with hydrocephalus caused by brain stem cavernoma. **A** : sagittal plane, **B** : axial plane, and **C** : coronal plane preoperative CT scan), and **D-E** : sagittal postoperative MRI-CSF study showing sufficient CSF flow through the ventriculostomy.

Seventeen of these patients underwent brain computed tomography scan and three of them had magnetic resonance imaging (MRI) brain as part of the preoperative planning. The diagnoses of the cause of hydrocephalus as well as the identification of the anatomical landmarks have both reduced the risk of complications.

In operating theater, the patients were positioned supine with head elevated ∼30 degrees fixed on Mayfield fixation system or lying on a horseshoe and a small area of the skin of the head was shaved. The incision was done ∼3 cm from the midline, along the midpupillary line ∼2 cm anteriorly of the coronal suture (Kocher point). The length of the incision was ∼5 cm and an 8 mm burr hole was performed with the usual technique. The appropriate use of the anatomical landmarks was of primary importance, as the procedure was performed without neuronavigation control. The endoscopic system used for these procedures is the Minop system (B. Braun, Germany) with a rigid endoscope from Aesculap, United States. The endoscope diameter was 6 mm and the sheath had four channels. The biggest one was used by the endoscope and the working channel was situated above the endoscope. Free hand technique was applied to introduce the endoscope into the lateral ventricle. Irrigation was performed using Ringer's solution and once lateral ventricle was inspected, then the endoscope was advanced through the Monro's foramen into the third ventricle, where it was possible to recognize the anatomical landmarks of the floor of the third ventricle (mammillary bodies and infundibular process). The fenestration of the roof was performed using bipolar diathermy following dilatation of the stoma by a balloon to widen further the fenestration of the floor of the third ventricle. The ETV was considered successful when there was complete resolution of the symptoms caused by hydrocephalus.

## Results

A total number of twenty patients were diagnosed with hydrocephalus and underwent ETV. Fourteen were male (70%) and six were female (30%). Their mean age was 56.5 years (median: 60.5 years and range: 19–79). The follow-up period ranged between 3 and 36 months. In two patients, ETV failed and had ventriculoperitoneal shunt done; the first was done 3 months after the ETV and the second one 12 months after the ETV. In both cases where the ETV failed, the patients were originally diagnosed with normal pressure hydrocephalus and underwent urgent shunt insertion after being transferred in acute and emergency department with low level of consciousness. Five patients presented with complications postoperatively ( Table 2 ).

**Table 2:** Post-ETV complications

	Causes	Age	Complications	Outcomes
**1**	Obstructive tumor—fourth ventricle	46	Claimed blindness not proven	Resolved in 24 h
**2**	Normal pressure hydrocephalus	66	III nerve palsy	Resolved in 3 mo
**3**	Obstructive cavernoma	62	Intraventricular hemorrhage	Neurologic deficit
**4**	Obstructive infection	41	Intracerebral hemorrhage	Neurologic deficit
**5**	Obstructive tumor—fourth ventricle	58	Weber syndrome	Neurologic deficit

Abbreviation: ETV, endoscopic third ventriculostomy.

The intracerebral hemorrhage was of small volume and it did not require surgical management. All patients after the initial procedure had postoperative MRI with cerebrospinal fluid flow sequence in 3 and 12 months.

## Discussion

The ETV is a minimally invasive reliable technique, which in future might represent the gold standard method in the management of hydrocephalus. When ETV was first introduced in neurosurgery, the primary indication was obstructive hydrocephalus. ETV is currently performed in patients suffering from nonobstructive hydrocephalus such as normal pressure hydrocephalus and trauma with promising results. Nevertheless, with regard to the managements of normal pressure hydrocephalus with ETV, there is no global consensus in the efficiency such method {Balevi:2017hj}{Tudor:2015hd}. As a result, the success rate of ETV varies from an overall 70 5 to 97% in the first year post-ETV, 6 while the complication rate remains low. 7

In our center, we had complications in five patients. Three presented with neural complications such as blindness, which was resolved in 24 hours; Weber syndrome, which persisted; and third nerve palsy, which was resolved after 4 weeks. Neural complications are well documented during the ETV procedure and have a low success rate (0.5–1.4%) especially those with persisting consequences. 8 The principal pathophysiologic mechanisms of these complications are either direct damage during the insertion of the endoscope or increased intracranial pressure caused by either an obstruction of the outflow channels or by excessive irrigation volume. We did not notice any direct injury in our patients nor malfunction of the irrigation system. Intraoperative hemorrhage was noticed in two patients. In the first case, the intraventricular bleeding was controlled and managed successfully using continuous irrigation and compression. The origin of that bleeding was most likely of venous origin caused by accidental tearing of the vein. The second patient with vascular injury suffered from intraparenchymal injury caused during the insertion of the endoscope. The bleeding was minor and no further neurosurgical procedure was required. This kind of bleeding is relatively frequent occurring in 16.5% of ETV procedures. 9 Major bleeding may also occur, especially in the event of injury of branches of basilar artery, but such a serious complication was noticed only in 0.49% of the cases. 10

In our series of cases, the ETV failed in two patients (11%). Failure is defined when symptoms of hydrocephalus persisted and further management was required with shunt insertion. Both patients with failed ETV were diagnosed with normal pressure hydrocephalus. In these cases, while shunt insertion represents the first choice of treatment, ETV is also performed but without definite benefits. Nevertheless, the perforated floor of the third ventricle that reduces the systolic intracranial through the stoma 11 as well as the fact that ETV is a minimally invasive technique has made it attractive in the treatment of nonobstructive hydrocephalus. In three of our patients with normal pressure hydrocephalus, ETV was successful. The main difference in comparison with the failed cases is the duration and the severity of the symptoms. Patients with short duration of symptoms, mild pollakiuria, and gait disturbance have better outcome when treated with ETV. 12

In our series of cases, performing ETV leads also to successful management of post-traumatic hydrocephalus. While there is lack of class I to IV studies regarding the efficacy of ETV in post-traumatic hydrocephalus, there is no contraindication either. 13 It is not possible to draw any safe conclusion by the mere case of one patient, but in addition to other cases reported in literature, we believe that ETV in post-traumatic brain injury should always be taken in consideration, while further trials are necessary to prove its benefit.

## Conclusions

ETV is a surgical technique that is based on bypassing the eventual obstacle of the cerebral cerebrospinal fluid flow. It is a minimally invasive procedure, relatively safe with good success rate.

In our center, in the last 3 years we have been performing ETV in patients suffering from hydrocephalus either obstructive or nonobstructive as part of routine management of the pathology. While we consider ETV a successful and safe technique for the patients suffering from hydrocephalus, we strongly believe that further studies are necessary to establish this procedure as treatment of choice for hydrocephalus.

## References

[1] Buxton N Ho K J Macarthur D Vloeberghs M Punt J Robertson I Neuroendoscopic third ventriculostomy for hydrocephalus in adults: report of a single unit's experience with 63 cases Surg Neurol 2001 55 02 74 78 11301084 10.1016/s0090-3019(01)00352-4

[2] Mixter W J Ventriculoscopy and puncture of the floor of the third ventricle Boston Med Surg J 1923 188 277 278

[3] Jones R F Stening W A Brydon M Endoscopic third ventriculostomy Neurosurgery 1990 26 01 86 91, discussion 91–92 2294483 10.1097/00006123-199001000-00012

[4] Drake J M Ventriculostomy for treatment of hydrocephalus Neurosurg Clin N Am 1993 4 04 657 666 8241788

[5] Beuriat P-A Puget S Cinalli G Hydrocephalus treatment in children: long-term outcome in 975 consecutive patients J Neurosurg Pediatr 2017 20 01 10 18 28430083 10.3171/2017.2.PEDS16491

[6] Vulcu S Eickele L Cinalli G Wagner W Oertel J Long-term results of endoscopic third ventriculostomy: an outcome analysis J Neurosurg 2015 123 06 1456 1462 26230473 10.3171/2014.11.JNS14414

[7] Cinalli G Springer International Publishing 2018 1 74

[8] Jung T-Y Chong S Kim I Y Prevention of complications in endoscopic third ventriculostomy J Korean Neurosurg Soc 2017 60 03 282 288 28490153 10.3340/jkns.2017.0101.014 PMC5426448

[9] Bouras T Sgouros S Complications of endoscopic third ventriculostomy J Neurosurg Pediatr 2011 7 06 643 649 21631203 10.3171/2011.4.PEDS10503

[10] Kawsar K A Haque M R Chowdhury F H Avoidance and management of perioperative complications of endoscopic third ventriculostomy: the Dhaka experience J Neurosurg 2015 123 06 1414 1419 26024001 10.3171/2014.11.JNS14395

[11] Hailong F Guangfu H Haibin T Endoscopic third ventriculostomy in the management of communicating hydrocephalus: a preliminary study J Neurosurg 2008 109 05 923 930 18976086 10.3171/JNS/2008/109/11/0923

[12] Balevi M Endoscopic third ventriculostomy in normal pressure hydrocephalus and symptomatic long-standing overt ventriculomegaly Asian J Neurosurg 2017 12 04 605 612 29114272 10.4103/ajns.AJNS_54_15 PMC5652084

[13] De Bonis P Tamburrini G Mangiola A Post-traumatic hydrocephalus is a contraindication for endoscopic third-ventriculostomy: isn't it? Clin Neurol Neurosurg 2013 115 01 9 12 22925601 10.1016/j.clineuro.2012.08.021

